# Baseline well-being, perceptions of critical incidents, and openness to debriefing in community hospital emergency department clinical staff before COVID-19, a cross-sectional study

**DOI:** 10.1186/s12873-020-00372-5

**Published:** 2020-10-15

**Authors:** Laura Cantu, Listy Thomas

**Affiliations:** Frank H. Netter MD School of Medicine, 370 Bassett Rd, North Haven, CT 06473 USA

**Keywords:** Debriefing, Peer support, Well-being, HADS, ProQOL, Critical incident, COVID-19, Secondary traumatic stress, Burnout

## Abstract

**Background:**

Emergency department personnel routinely bear witness to traumatic experiences and critical incidents that can affect their own well-being. Peer support through debriefing has demonstrated positive impacts on clinicians’ well-being following critical incidents. This study explored community hospital emergency department staff’s perceptions of critical incidents, assessed openness to debriefing and measured baseline well-being. Our analysis provides a baseline of provider well-being immediately prior to the local onset of COVID-19. The potential need for additional resources to support frontline providers during the pandemic can be evaluated.

**Method:**

We conducted a cross-sectional study for 4-weeks prior to the first COVID-19 case in Connecticut using a survey offered to an interprofessional group of emergency department clinical staff. The main outcome measures were the Hospital Anxiety and Depression Scale (HADS) and the Professional Quality of Life (ProQOL) scale. Pearson’s chi-square test was used to identify significant differences in perceptions of critical incidents and debriefings between professional categories. One-way ANOVA and Tukey’s test were used to analyze significant differences in well-being between professional categories.

**Results:**

Thirty-nine clinical personnel from St. Vincent’s Emergency Department responded to the survey. Events frequently selected as critical incidents were caring for critically ill children (89.7%), mass casualty events (84.6%), and death of a patient (69.2%). Critical incidents were commonly reported (81.6%) as occurring once per week. Additionally, 76.2% of participants reported wanting to discuss a critical incident with their team. Across all respondents, 45.7% scored borderline or abnormal for anxiety, 55.9% scored moderate for burnout, and 55.8% scored moderate to high for secondary traumatic stress.

**Conclusions:**

At baseline, providers reported caring for critically ill children, mass casualty events, and death of a patient as critical incidents, which typically occurred once per week. Death of a patient occurs at increased frequency during the protracted mass casualty experience of COVID-19 and threatens provider well-being. Receptiveness to post-event debriefing is high but the method is still underutilized. With nearly half of staff scoring borderline or abnormal for anxiety, burnout, and secondary traumatic stress at baseline, peer support measures should be implemented to protect frontline providers’ well-being during and after the pandemic.

## Background

Emergency department clinical staff manage traumatic events as a routine part of their careers in medicine. Although these staff members have not experienced the patient’s trauma first-hand, the strong emotional reactions providers may feel following caring for patients who have experienced such events can affect them in many ways; including cognitively, behaviorally, emotionally, and physically [1–4]. As a result, frontline healthcare workers are at risk for secondary traumatic stress responses that range from exhaustion and avoidance to hypervigilance, physical illness, and presenteeism [5, 6]. At baseline, healthcare workers report high levels of burnout, secondary traumatic stress, and suicidal ideation, which are correlated to decreases in performance and patient care [2, 7–11]. The new demands placed on providers by COVID-19 will likely negatively impact providers’ mental health [12, 13]. An earnest assessment of the utilization of stress mitigation strategies must be conducted for the protection of frontline providers.

Discussion-based stress interventions in real time (in situ) have been developed to improve peer support well-being and providers’ ability to return to work, while reducing stress manifestations [14, 15]. Critical incident stress debriefing (CISD), an older and more structured form of debriefing, has come under scrutiny for possibly worsening PTSD symptoms or secondary traumatic stress. Notably, the literature that suggests these adverse outcomes included studies which used CISD outside the model’s intended framework, such that debriefing occurred one-on-one with patients instead of in a group setting with frontline providers [16, 17]. Newer methods of post-event debriefing, such as the INFO and DISCERN models, [18, 19] are structured to avoid many of the perceived pitfalls of CISD through their more immediate timing and provider-implemented style [20]. In this paper, the term “debriefing” will be used to indicate a form of peer support, discussion-based stress intervention.

These low resource methods have demonstrated efficacy for interprofessional staff following pre-selected clinical events or critical incidents [21–24]. A critical incident has been defined by Magyar et al as, “a self-defined traumatic event that causes individuals to experience such strong emotional responses that usual coping mechanisms are ineffective” [25]. Pediatric and adult resuscitations are common pre-selected clinical events which should initiate a debriefing [14, 19, 24, 26]. Clinical events previously recognized as distressing for staff or cited as critical incidents in the literature include death of a patient, multi-trauma, and death of young patients [1, 19, 27]. To cope with emotionally challenging patient cases, nurses have previously reported reliance upon peer support and physicians have voiced a preference for a more formal support structure [22, 23, 26].

The aim of this study is to describe the well-being of community hospital emergency department clinical staff immediately prior to the local onset of COVID-19 and identify their perceptions surrounding critical incidents and post-event, discussion-based interventions. The current low level of hospital instituted peer support programs and current risks to health professionals’ well-being needs to be addressed [28]. By demonstrating healthcare workers’ openness to peer support, this study serves as an evidence-based call to action for the implementation of in situ debriefing to mitigate stress impacts during the pandemic.

## Methods

### Study design

This cross-sectional study was conducted in the 4-weeks immediately prior to the announcement of the first COVID-19 positive case in a Connecticut state resident [29]. The study site was a community hospital emergency department with no formal peer support debriefing program, akin to most community hospitals [27, 30]. Survey distribution began in February 2020 and responses were collected through early March 2020. The study used self-report questionnaires in Qualtrics, a secure HIPAA compliant HITRUST certified survey software published by Qualtrics, to collect demographic data from the interprofessional group of respondents. The survey questions collected staff’s experiences and perspectives surrounding critical incidents and post-event discussion-based interventions. Levels of anxiety, depression, burnout, compassion satisfaction, and secondary traumatic stress were measured using validated clinical questionnaires and scoring scales, described below in more detail. All survey responses were collected anonymously.

### Study participants

The online survey was distributed through a department-wide e-mail to all emergency department clinical staff at a community hospital in Connecticut. Study participants encompassed the following clinical roles: registered nurses (RN), physician assistants (PA), physicians, resident physicians, and emergency department technicians (ED Tech).

### Demographic data

Demographic data collected from the participants included gender, clinical role, and years of practice.

### Critical incidents

Participants were given the following definition for a critical incident prior to responding to questions regarding their experiences and perspectives, “A critical incident is a self-defined traumatic event that causes individuals to experience such strong emotional responses that usual coping mechanisms are ineffective.”

### Hospital anxiety and depression scale (HADS)

The HADS was used to measure the levels of anxiety and depression in the medical staff [31–33]. The HADS questionnaire contained 14 items, seven items relevant to each anxiety and depression, graded for experience within the past 7 days. Each item was scored between 0 and 3 and the scores within each category were totaled for a cumulative score ranging 0–21. Higher scores indicate more abnormal levels of anxiety and depression.

### Professional quality of life (ProQOL)

The ProQOL version 5 assessment was used to measure the levels of compassion satisfaction, burnout, and secondary traumatic stress in the medical staff [34, 35]. The ProQOL is a five-point Likert scale consisting of 30 items. Scores cumulate within each of the three outcome measures for a total score ranging from 10–50. Higher scores indicate higher levels of compassion satisfaction, burnout, and secondary traumatic stress.

The ProQOL defines measures in the following ways, “Compassion satisfaction is about the pleasure you derive from being able to do your work well” [36]. Secondary traumatic stress is the development of emotional duress due to indirect exposure to trauma derived while helping others [37].

### Statistical analysis

For categorical variables, including demographic data and self-report data regarding critical incidents, proportions were reported. Pearson’s chi-square test was used to further analyze for associations between professional categories and responses to critical incident and debriefing questions. Due to the small sample size, the registered nurse and physician assistant professional categories were collapsed into a single group. Outcomes of the HADS and ProQOL scales were reported as proportions and one-way ANOVA was employed to assess for differences between mean well-being scores and demographic groups. Further analysis of statistically significant findings was conducted with Tukey’s post hoc test. All *P*-values were two-sided and, if below 0.05, the results were considered statistically significant. Analyses were conducted using IBM SPSS Statistics for Windows Version 26 (IBM Corp., Armonk, N.Y., USA).

## Results

### Participants

In total, 46 people consented to the survey. For this study, seven respondents who did not respond to all questions or returned the survey after the first positive COVID-19 local case were excluded, leaving a sample of 39. This sample represents 32.5% of the emergency department’s 120 employees.

A total of 39 staff members (6 physicians, 27 combined registered nurses and physician assistants, and 6 emergency department technicians) completed the demographics and opinion portion of the survey, 35 staff members (5 physicians, 24 registered nurses and physician assistants, and 6 emergency department technicians) completed the HADS, and 34 staff members (4 physicians, 24 registered nurses and physician assistants, and 6 emergency department technicians) completed the ProQOL. The demographic data of the participants are shown in Table 1.

**Table 1:** Demographic data represented in the sample

Variable	Number	Percent
**Gender**		
Female	29	74.4%
Male	10	25.6%
**Clinical Role**		
Physician	6	15.4%
RN/PA	27	69.2%
ED Tech	6	15.4%
**Years of Practice**		
< 3 years	4	10.3%
3–10 years	16	41.0%
11–20 years	7	17.9%
20+ years	12	30.8%

### Critical incidents

**Fig. 1:**
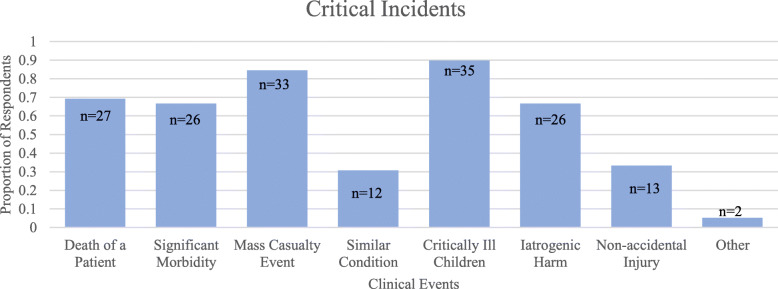
Proportion of respondents, out of 39, who considered each clinical event a critical incident

The clinical event most commonly selected as a critical incident was “caring for a critically ill child” by 89.74% (*n* = 35) of respondents. Frequency of clinical events considered critical incidents are shown in Fig. 1. The “Other” category consisted of 2 respondents who wrote in “death of a child.”

The proportion of respondents who selected each clinical event are shown by professional category in Table 2. Pearson’s chi-square analysis was used to identify the associations between clinical role or years of practice and clinical events considered critical incidents. There was a statistically significant association between selection of mass casualty event as a critical incident and clinical role (χ(1) = 6.850, *p* = .033), with 50% selection amongst emergency department technicians compared to 83.3% of physicians and 92.6% of registered nurses and physician assistants. There was no statistically significant association between any clinical events and years of practice.

**Table 2:** Clinical events selected as critical incidents by professional category

Professional category	Death of a patient	Injury resulting in significant morbidity	Mass casualty event	Patient with same condition as provider	Critically ill child	Iatrogenic harm	Non-accidental injury
**Clinical Role**							
Physician	66.7% (n = 4)	83.3% (*n* = 5)	83.3% (*n* = 5)	33.3% (n = 2)	100% (*n* = 6)	66.7% (n = 4)	50% (*n* = 3)
RN/PA	70.4% (*n* = 19)	63% (*n* = 17)	92.6% (n = 25)	25.9% (n = 7)	85.2% (*n* = 23)	70.4% (n = 19)	25.9% (n = 7)
ED Tech	66.7% (n = 4)	66.7% (*n* = 4)	50% (n = 3)	50% (*n* = 3)	100% (n = 6)	50% (n = 3)	50% (n = 3)
**Practice Years**							
< 3 years	75% (n = 3)	75% (n = 3)	75% (n = 3)	50% (n = 2)	100% (n = 4)	50% (n = 2)	50% (n = 2)
3–10 years	62.5% (*n* = 10)	62.5% (n = 10)	75% (*n* = 12)	25% (n = 4)	81.3% (*n* = 13)	68.8% (*n* = 11)	18.8% (n = 3)
11–20 years	85.7% (n = 6)	71.4% (n = 5)	85.7% (n = 6)	28.6% (n = 2)	100% (n = 7)	71.4% (n = 5)	42.9% (n = 3)
20+ years	66.7% (*n* = 8)	66.7% (n = 8)	100% (n = 12)	33.3% (n = 4)	91.7% (n = 11)	66.7% (n = 8)	41.7% (n = 5)

The proportion of participants who reported participating in a critical incident during the last 12 months was 97.4% (*n* = 38) and the most common frequency for critical incidents was reported as “once per week” by 81.6% (*n* = 31) of respondents (Table 3).

**Table 3:** Reported frequency of experiencing a critical incident within the last 12 months

Frequency of CI	Number	Percent
Once per week	31	81.6%
Multiple times per week	5	13.2%
Multiple times per shift	2	5.3%

### Openness to debriefings

**Fig. 2:**
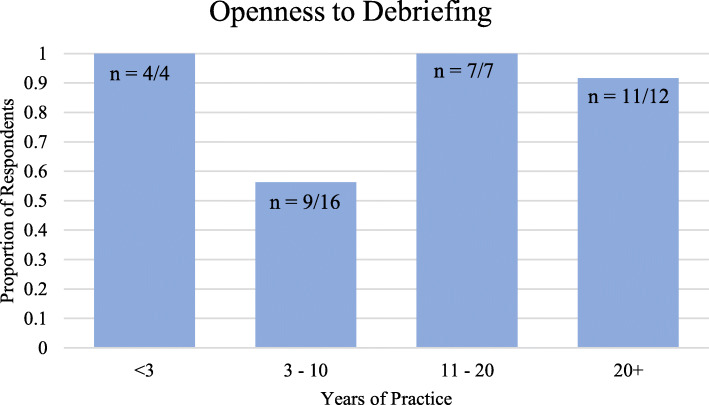
Proportion of respondents, by years of practice, who reported wanting to discuss a critical incident with their team

The proportion of respondents who reported having discussed a critical incident with their team during the past 12 months was 64.1% (*n* = 25) and 100% (n = 25) of those respondents reported finding it useful to their well-being. Of all respondents, 79.5% (*n* = 31) reported wanting to discuss a critical incident with their team in the past 12 months. There was a statistically significant association between desire to discuss a critical incident and years of practice (χ(1) = 9.229, *p* = .026), with the highestproportion from the < 3 years of practice (100%, *n* = 4) and 11–20 years of practice (100%, *n* = 7) groups and the lowest proportion amongst the 3–10 years of practice group (56.3%, *n* = 9) as shown in Fig. 2. There was no statistically significant association between wanting to discuss a critical incident and clinical role (χ(1) = 0.725, *p* = .696).

### Baseline well-being

The levels of anxiety and depression as measured by the HADS and levels of compassion satisfaction, burnout, and secondary traumatic stress as measured by the ProQOL have been reported in Table 4.

**Table 4:** Overall results of the HADS and ProQOL scales

**HADS**	Normal	Borderline abnormal	Abnormal	Mean	Std. Deviation
Anxiety	54.3% (*n* = 19)	25.7% (*n* = 9)	20.0% (*n* = 7)	7.20	3.954
Depression	85.7% (*n* = 30)	11.4% (*n* = 4)	2.9% (*n* = 1)	3.37	3.040
**ProQOL**	Low	Moderate	High	Mean	Std. Deviation
Compassion satisfaction	0% (*n* = 0)	64.7% (*n* = 22)	35.3% (*n* = 12)	39.44	6.165
Burnout	44.1% (*n* = 15)	55.9% (*n* = 19)	0% (*n* = 0)	23.47	6.278
Secondary traumatic stress	44.1% (*n* = 15)	52.9% (*n* = 18)	2.9% (*n* = 1)	23.24	6.867

There was a statistically significant difference between clinical roles for mean secondary traumatic stress scores as determined by one-way ANOVA (F (2, 31) = 5.811, *p* = .007). A Tukey post-hoc test revealed that secondary traumatic stress was statistically significantly lower in the combined RN/PA group (21.46 ± 6.043) compared to ED Techs (30.83 ± 6.369, *p* = .011). There was no statistically significant difference between the physicians and ED Techs (*p* = .115) or registered nurses and physician assistants (*p* = .987). There was no statistically significant difference between the gender or years of practice groups for mean secondary traumatic stress scores or between any groups for the remaining HADS and ProQOL measures.

## Discussion

### Critical incidents

Prior to the local onset of COVID-19 in Connecticut, the majority of frontline providers in this study identified caring for critically ill children (89.7%), mass casualty events (84.6%), and death of a patient (69.2%) as critical incidents that would render usual coping mechanisms ineffective. These findings are consistent with a descriptive, cross-sectional study conducted in 13 pediatric emergency departments across Australia and New Zealand, which found 81% of senior nurses and physicians believed death of a patient was a critical incident warranting debriefing [27]. At baseline, critical incidents were predominantly reported at a frequency of only once per week by 81.6% of providers. Perceptions of what clinical events are critical incidents may also be impacted in the post-COVID-19 landscape. Specifically, “caring for a patient with a condition that you or a loved one has,” which had the lowest respondent selection (30.8%), may increase in prevalence due to the potential development of fear amongst healthcare providers regarding contracting or transmitting COVID-19 to family members.

### Openness to debriefings

The majority of participants indicated a desire to discuss a critical incident with their team in the past 12 months, demonstrating a receptiveness to in situ stress interventions like post-event discussions or debriefings. There was a statistically significant difference between the proportion of providers who wanted to discuss a critical incident across years of practice; with the 3–10 years of practice group reporting the lowest proportion (56.3%), compared to 100% of providers with < 3 and 11–20 years of practice or 91.7% of providers with 20+ years of practice. The lower rate among mid-career providers may be attributed to self-perceptions of resilience. These outcomes reveal an opportunity for specific programming aimed at normalizing and promoting peer support among providers with 3–10 years of practice. Given the high rate of receptiveness across providers with more years of practice, this may also create a channel for senior mentorship to improve receptiveness across junior mentees.

There was no difference in openness to post-event discussion across clinical roles, further indicating potential for high uptake by interprofessional teams. Previous studies conducted in pediatric emergency department nurse populations have reported similar preferences for peer based support following critical incidents [22]. Given the potential use of travel nurses and outside medical providers to supplement hospital staffing, easily self-implemented, low-resource debriefings may provide foundation for building peer support amongst less familiar teams.

### Baseline well-being

Roughly half of all medical workers surveyed experienced borderline or abnormal anxiety (45.7%), moderate burnout (55.9%), or moderate to high secondary traumatic stress (55.8%). This level of burnout is consistent with previously reported levels of physician burnout. A call to action by the Massachusetts Medical Society in 2019 already considered the state of physician well-being a public health crisis [9]. Healthcare workers, who may face disillusionment as a result of this pandemic, would benefit from wider accessibility to various forms of stress interventions [38].

To our knowledge, this study is the first to report baseline well-being, opinions of critical incidents, and openness to debriefing amongst emergency department clinical staff immediately prior to the local onset of COVID-19. Several limitations of the study should be noted. The age of respondents was not collected in demographic data, although it would have been interesting to correlate the age of respondents with their markers of well-being. The small sample size collected at a single site reduces the power to detect group differences and the ability to generalize from the findings. Additionally, nonresponse bias may have influenced our findings. Providers who completed surveys may have had stronger feelings regarding mental health support compared to providers who declined, resulting in an unrepresentative sample. Baseline well-being may differ for emergency departments with institutionalized peer support programs.

At present, a longitudinal study aims to capture weekly impacts from critical incidents within this same population in order to assess how evolving COVID-19 cases manifest as critical incidents and effect provider well-being through time. Future plans include reassessment of HADS, ProQOL, and perceptions of critical incidents and debriefings after COVID-19 cases decrease to directly assess the impact of the pandemic on providers. Hospitals should also aim to collect local data on provider well-being, implement post-event stress debriefings, and assess stress mitigation interventions for efficacy.

## Conclusion

Our cross-sectional study of community hospital emergency department clinical staff in one institution found providers considered mass casualty events and death of a patient to be critical incidents. At baseline, providers experienced levels of anxiety, burnout, and secondary traumatic stress at levels previously identified as detrimental to personal and public health. All respondents who had discussed a critical incident with their team found this experience useful to their wellbeing. The majority of providers, including those with no prior debriefing experience, reported a desire for post-event, team-based discussions. The absence of institution supported debriefings and peer support groups in community hospitals is an area for improvement and discussion-based stress reduction interventions could be included in COVID-19 response trainings to promote peer support and frontline provider well-being.

This study was conducted prior to the onset of the pandemic, and in light of the likelihood of increased negative mental health impacts from COVID-19, this data may indicate a particularly vulnerable position for providers. Acute and potentially enduring moral injury sustained from the potential shortages of appropriate proper protective equipment (PPE), lack of evidence based data to inform decision making, crisis standards of care, and the trauma of witnessing large numbers of individuals experiencing serious illness and death in the absence of family may all contribute to high levels of stress among frontline healthcare providers during and after the pandemic. These experiences are compounded by the stress of social isolation and lack of appropriate human interactions that would otherwise mitigate stress. Researchers will likely find significant impairments in anxiety, burnout, and secondary traumatic stress as the lingering effects of COVID-19 effect frontline workers. Peer support measures that facilitate debriefings should be implemented to protect frontline providers’ well-being during and after the pandemic.

## Supplementary information

**Additional file 1.** Table: Survey questions

## Data Availability

The datasets used and/or analyzed during the current study are available from the corresponding author on reasonable request.

## Abbreviations

HADS: Hospital anxiety and depression scale
ProQOL: Professional quality of life
ED Tech: Emergency department technician
RN: Registered nurse
PA: Physician assistant
COVID-19: Coronavirus virus disease 2019
