# Supplementary material for: Baseline well-being, perceptions of critical incidents, and openness to debriefing in community hospital emergency department clinical staff before COVID-19, a cross-sectional study
Source: BMC Emerg Med. 2020 Oct 15;20:82. doi: 10.1186/s12873-020-00372-5 (PMC7558255; doi:10.1186/s12873-020-00372-5)
Supplement: Supplementary file 1 — Additional file 1. Table: Survey questions [file 12873_2020_372_MOESM1_ESM.docx]

*Table:* Survey Questions

|  |
| --- |
| 1. Gender  - Female/Male/Other |
| 1. Clinical Role  - Attending Physician/ED Tech/EMS/Medical Student/Nursing Student/Pharmacist/ Physician Assistant/Physician Assistant Student/Registered Nurse/Resident Physician/ Unit Secretary/Other |
| 1. Years of Practice  - < 1/ 1–3/ 3–5 / 5–10/ 10–15/ 15–20/ 20+ |
| 1. Which of the following events would you consider a critical incident? (Choose all that apply.)  - Death of a patient/ Injury resulting in significant morbidity/ Mass casualty events/ Caring for a patient with a condition that your or a loved one has/ Caring for a critically ill child/ An event that may have resulted in iatrogenic harm to the patient under your care/ Non-accidental injury/ Other |
| 1. In the past 12 months, have you participated in a patient care scenario that was a critical incident?  - Yes / No |
| 1. On average, how often do you participate in a patient care scenario that you would label as a critical incident?  - Once per week/ Multiple times per week/ Once per shift/ Multiple times per shift |
| 1. Did your involvement in this critical patient care scenario negatively impact your well-being?  - Yes / No |
| 1. In the past 12 months, have you wanted to discuss a critical incident with your team?  - Yes / No |
| 1. If yes to question 8: Did you find it useful to your well-being to discuss with your team?  - Yes / No |
| 1. In the past 12 months, did you discuss a critical incident with your team?  - Yes / No |
